# Automated collection and categorisation of STM images and STS spectra with and without machine learning

**DOI:** 10.3762/bjnano.16.99

**Published:** 2025-08-18

**Authors:** Dylan Stewart Barker, Adam Sweetman

**Affiliations:** 1 School of Physics and Astronomy, University of Leeds, Leeds LS2 9JT, United Kingdom

**Keywords:** automated, machine learning, spectroscopy, scanning tunnelling microscopy (STM), scanning tunnelling spectroscopy (STS)

## Abstract

Atomic resolution scanning probe microscopy, and in particular scanning tunnelling microscopy (STM) allows for high-spatial-resolution imaging and also spectroscopic analysis of small organic molecules. However, preparation and characterisation of the probe apex in situ by a human operator is one of the major barriers to high-throughput experimentation and to reproducibility between experiments. Characterisation of the probe apex is usually accomplished via assessment of the imaging quality on the target molecule and also the characteristics of the scanning tunnelling spectra (STS) on clean metal surfaces. Critically for spectroscopic experiments, assessment of the spatial resolution of the image is not sufficient to ensure a high-quality tip for spectroscopic measurements. The ability to automate this process is a key aim in development of high resolution scanning probe materials characterisation. In this paper, we assess the feasibility of automating the assessment of imaging quality, and spectroscopic tip quality, via both machine learning (ML) and deterministic methods (DM) using a prototypical tin phthalocyanine on Au(111) system at 4.7 K. We find that both ML and DM are able to classify images and spectra with high accuracy, with only a small amount of prior surface knowledge. We highlight the practical advantage of DM not requiring large training datasets to implement on new systems and demonstrate a proof-of-principle automated experiment that is able to repeatedly prepare the tip, identify molecules of interest, and perform site-specific STS experiments using DM, in order to produce large numbers of spectra with different tips suitable for statistical analysis. Deterministic methods can be easily implemented to classify the imaging and spectroscopic quality of a STM tip for the purposes of high-resolution STM and STS on small organic molecules. Via automated classification of the tip state, we demonstrate an automated experiment that can collect a high number of spectra on multiple molecules without human intervention. The technique can be easily extended to most metal–adsorbate systems and is promising for the development of automated, high-throughput, STM characterisation of small adsorbate systems.

## Introduction

Scanning tunnelling spectroscopy (STS) extends the capability of scanning tunnelling microscopy (STM) beyond topographic imaging, allowing for the direct measurement of the electronic properties of surfaces and molecules with atomic precision. This opens up the ability to map the local density of states (LDOS) of a sample with high spatial resolution [1–3]. Peaks within a map of the LDOS correspond to increases in conductance at specific bias values, revealing the energy levels of key features (e.g. molecular orbitals in the case of molecular samples) within the material.

As for STM imaging, the sharpness and overall tip shape is crucial in optimising the spatial resolution of STS measurements; sharp tips result in localised tunnelling through a single position, whereas blunt or misshaped tips cause averaging of contributions over larger areas, reducing the spatial resolution and potentially blending the electronic features between different sites. However, even for tips with high spatial resolution, different tip structures and probe terminations are known to influence these results [4–7].

STS measurements are the result of an integration over the available density of states (DOS) in both the tip and the sample, with the current measured therefore being proportional to the convolution of two. To isolate the DOS of the sample, it is crucial that the tip has a nominally “flat” DOS, which is typically achieved by using a purely metallic tip. However, most tips do not demonstrate a perfectly flat local density of states (LDOS) as they have a complex electronic structure governed by the geometry of the metallic cluster at the tip apex [8–12]. Non-metallic contaminants can also strongly perturb the electronic structure of the tip.

Methods of optimising the probe state for ideal STS are slow and laborious, involving indentation into a metal surface and bias pulses applied to the tip, manually checking spectra and imaging after each probe shaping attempt. The automation of this process could result in a more rapid and reproducible method for performing spectroscopy measurements.

To classify the state of the probe for STS experiments, spectra are usually taken over bare areas of a metallic substrate. On coinage metal surfaces, these 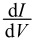 spectra typically exhibit a characteristic feature corresponding to the surface state, which appears as a step function around a specific bias value, which for the Au(111) surface appears at around −0.48 V [13–14].

One notable attempt to automate this classification using machine learning (ML) was carried out by Wang et al. [15]. This work aimed to classify the state of a STM tip based on STS measurements of the Au(111) surface. Using a total of 1789 archived 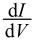 spectra, a ML model was trained which aimed to classify new spectra into one of five categories, based on the similarity of each the spectrum to an idealised surface state.

This schema achieved final precision in classification of 84% and a recall of 74%. Similarly to image classification in scanning probe microscopy (SPM) [16–18], the availability of such a large amount of data for training is usually very low, making ML-based classifiers troublesome to train. In addition to the lack of data, ML models require careful labelling and a high level of knowledge from the labeller to be able to train such a model. Switching to a new substrate system is likely to require retraining of the model, and furthermore, even after a successful training, it is still often unclear what the model is learning from the input data, a problem which leads to these models being referred to as a “black box”. Because of these limitations, there is a strong case to develop methods which do not rely on ML, circumventing these drawbacks whilst still being able to make precise classifications of the tip quality for use in automation.

**Figure 1:**
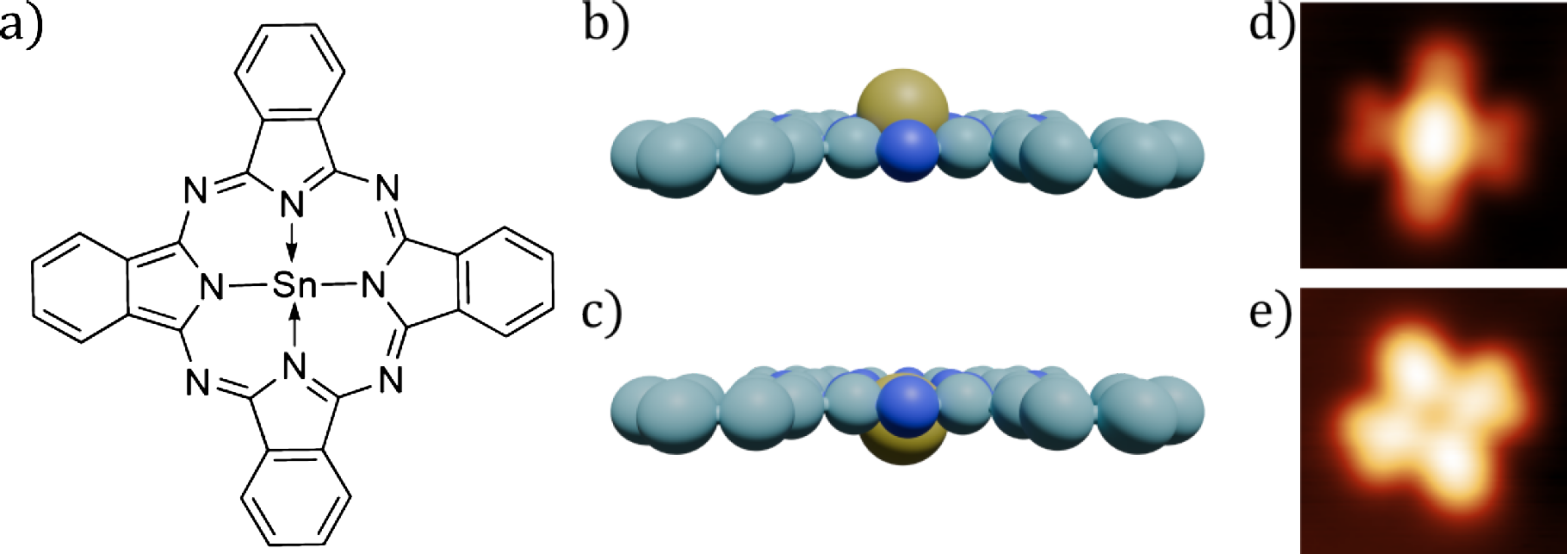
(a) Structure of tin phthalocyanine (SnPc). Side-on view of SnPc, illustrating its non-planar nature in the (b) SnUp and (c) SnDown configurations. (d) and (e) show constant-current STM images of the SnUp and SnDown configurations respectively, taken at 5 K, −100 mV, 50 pA.

In the following work, we use the prototypical system of tin phthalocyanine (SnPc) on Au(111) to investigate the feasibility of a DM automated classifier and compare it to ML methods. This molecular system has the advantage that the SnPc adsorbs on the surface in two distinct configurations, one with the tin atom facing up (SnUp), and the other with the tin facing down (SnDown) (Figure 1), providing a variety of molecular configurations to challenge the automated molecular identification.

In addition to the classification of the probe quality based on the surface state, we use a DM-based cross-correlation (CC) feature finding method [19] in order to also assess the imaging quality of the tip, and also automatically locate various molecules on the surface. Combining these methods, it is possible to conduct a fully automated experiment, where a large number of STS measurements can be obtained over various molecules automatically with optimised tips and the quality of the spectra and image assessed automatically.

## Methods

### Experimental details

We used a third-generation commercial low-temperature (LT) STM NC-AFM instrument (Scienta Omicron GmbH), which was operated using an RC5 Nanonis controller, with all experiments carried out in UHV (base pressure ≤5 × 10^−11^ mbar) cooled to 5 K. Gold and silver crystals (spl.eu) were prepared via repeated sputter–anneal cycles, sputtering under an argon pressure of ≈5 × 10^−5^ mbar, with a beam energy of 1.5 kV for 30 min, measuring a drain current of ≈7.0 μA, before annealing at 500 °C for 30 min and then placed into the scan head for imaging. Platinum–iridium STM tips were used throughout this work and were prepared by standard STM methods (voltage pulses, controlled contacts with the sample) until good atomic resolution was obtained in STM feedback.

SnPc was deposited onto the Au(111) surface using a custom-built evaporator, where the powdered source material is contained within a glass crucible using glass wool, around which a coil of tantalum wire is wound, providing a source of heat for the crucible. The target temperature for SnPc deposition was 360 °C; once reached, the cryostat shields were opened for 1 h, before closing and checking the coverage in STM. Once deposited, the sample was cold annealed to room temperature, which has the effect of driving the molecules preferentially to the “elbow” sites of the herringbone structure.

An STS spectrum (differential conductance) can be obtained in practice using one of two methods. Both begin by positioning the STM tip at a desired lateral position on the surface whilst scanning in STM feedback. At this point, the feedback loop is disabled, keeping the tip–sample distance constant throughout the spectroscopy measurement. The voltage is then swept through a range of values whilst measuring the current, which is obtained as a function of the varying voltage, *I*(*V*). This curve can then be differentiated with respect to the voltage to obtain the differential conductance, 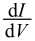, spectra.

Alternatively, the derivative signal, 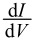, as a function of voltage, can be directly measured using the lock-in technique. In this method, an AC signal is generated by applying a small modulation voltage, *V*_M_cos(ω*t*), to the bias. Due to this modulation, the measured current is expressed as


[1] $$I\left(t\right)=f\left(V+V_{\text{M}}\cos\left(\omega t\right)\right),$$

where *V*_M_ is the modulation amplitude and ω is the frequency. Applying this modulation around a central voltage creates a corresponding modulation in the measured tunnel current with an amplitude proportional to the gradient of the 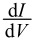 curve at that bias. Therefore, once the tip is in position, the bias can be swept through a range whilst applying the modulation. The resultant current can then be detected by a lock-in amplifier, where its amplitude for small values of *V*_M_ is proportional to 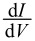, therefore directly measuring the differential conductance of the sample. Throughout the work presented here, the conductance was measured directly using the lock-in technique.

## Results

To create the ML-based classification models needed for this work, a large amount of data was needed in the form of STS spectra taken with a variety of different tip shapes and configurations. The dataset generation procedures were created using LabVIEW, which interfaces directly with the Nanonis controller. The process of the dataset generation was performed in a manner similar to that described in Barker et al. [19], with some minor alterations, as described below.

**Figure 2:**
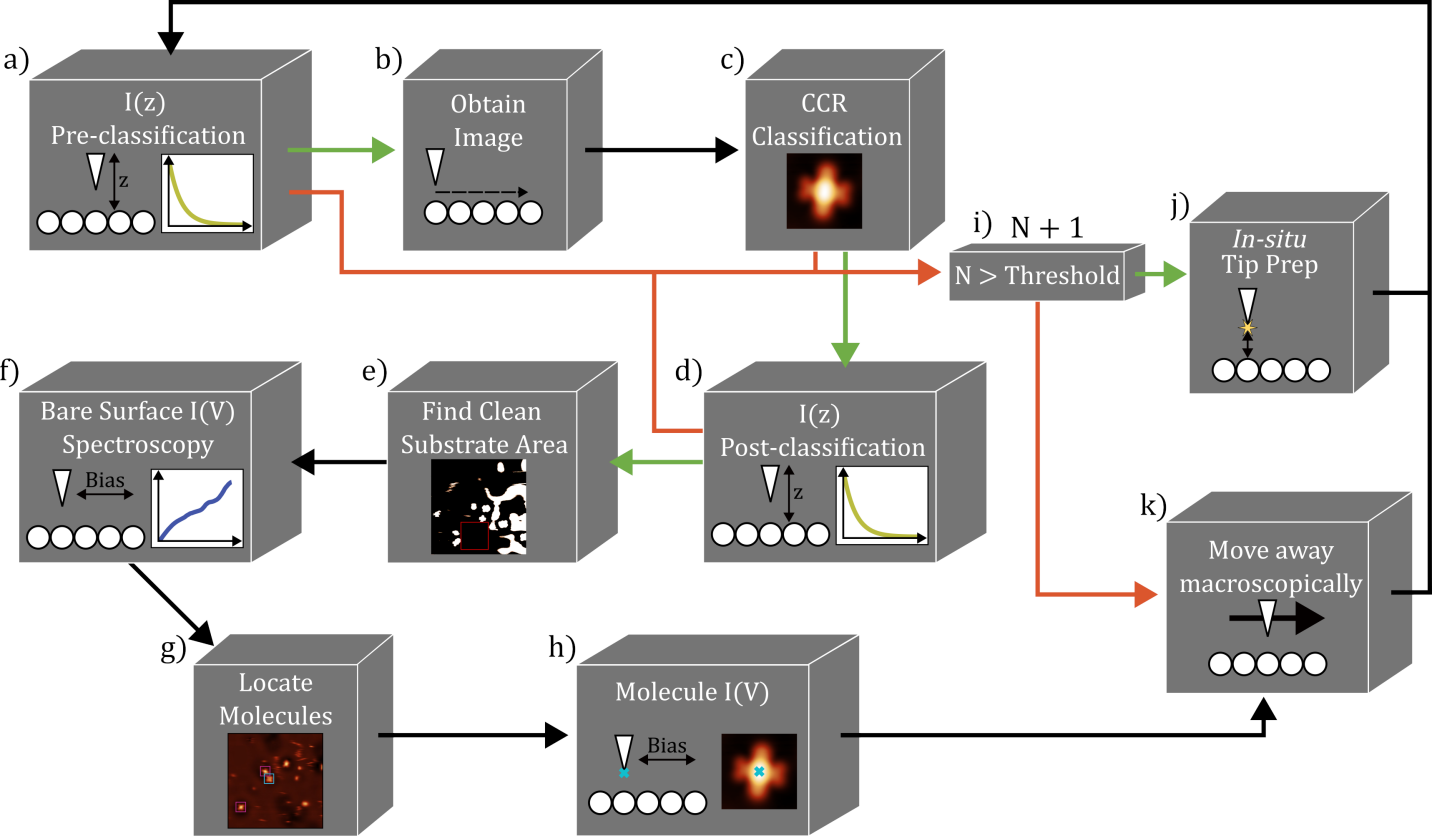
Schematic for the automated data gathering script. The script starts by taking an initial *I*(*z*) spectrum, (a), which is classified based on its exponential dependence. If the *I*(*z*) is classified as “good”, the script then obtains a scan of a specific area, (b), followed by a CC-based classification, (c). If the CC image classification determines the tip to be “good”, the script moves onto another *I*(*z*) classification, (d), followed by an analysis step to find a clean substrate area, (e). Using the area found in (e), the script obtains 15 *I*(*V*) spectra over different positions, (f). The script then locates the different configuration of molecules present in the scan, (g), before obtaining *I*(*V*) spectra over the centre positions of each molecule, (h). In the classification steps (a), (c), and (d), if the tip is classified to be “bad”, the script will move on to either an in situ tip preparation step, (j); if the number of shaping attempts without a “good” tip has exceeded a pre-determined threshold, (i), the tip is moved away macroscopically, (k), under the assumption that the area is not suitable for classification. Throughout the schematic, green arrows show positive classifications and red arrows indicate negative classifications.

The process of the automated dataset generation is shown in Figure 2. One addition to this method compared to the automated data gathering method described in Barker et al. [19] is the addition of *I*(*z*) classifications prior to performing imaging to ensure a tunnelling junction. This acts as a rapid “pre-filtering” step, eliminating tips that do not show a stable tunnelling junction (and hence are not suitable for STS) without the need to perform a complete image to characterise the tip. The classification of the state of the probe based on imaging is performed via the CC method as a key feature of a “good” tip for STS is also the sharpness of the probe, in order to ensure high spatial resolution in the acquired data. Further details on the CC and *I*(*z*) classification as implemented for the SnPc models is provided in the online supporting information.

Once the imaging classification is complete, the *I*(*z*) classification is performed again to check that a tip change did not occur during the scan. The obtained topograph is then analysed to find both a large area of clean metal substrate, over which *I*(*V*) spectra can be obtained, and to find the location of the molecules in various configurations, over which additional *I*(*V*) spectra are taken.

After completing this data gathering step, the tip moves away from the imaging area for a tip preparation event, in order to change the apex substantially before repeating the entire process to collect another dataset with a different tip. Throughout, if the tip is classified as “bad” in either of the *I*(*z*) classifications or the CC-based imaged classification, the script moves onto a tip preparation event. If the tip fails in being classified as “good” more than a set number of times in a row, the tip is moved away macroscopically, under the assumption that the current area of the surface is not suitable for classification; this typically occurs due to the area being damaged from prior tip preparation, or the absence of an SnUp molecule in the frame which is used in the CC classification of the image.

The CC classification is carried out as described in Barker et al. ([19]), with the reference image used being a cropped image of an SnUp molecule as is shown in Figure 1d. SnUp molecules were chosen for classification as in this configuration, the Sn atom in the molecule presents a higher aspect ratio than in the SnDown configuration, and so is more sensitive to the sharpness of the tip. Using this method with a threshold of *>*0.98, the model was able to reliably generate and identify sharp tips.

### Dataset summary

Using the data generation method described above, we obtained a total of 2604 individual spectra on the bare Au(111) surface, 86 of which were used for our classification models. In order to use this data for training and evaluation of models, the dataset required labelling. We note that labelling of the dataset is non-trivial as for ML models the model can only attempt to learn to evaluate spectra based on the ground truth provided by the labelling.

Labelling was carried out using a similar process to [19]. A custom Python script was written with a graphical interface. The script would show each spectrum individually, with a choice of four labels depending on the visibility of the surface state (SS): SS “good”, SS “step visible”, SS “peak visible”, and SS “not visible”. When classifying the data, the region around the surface state step was focused on, with the “good” label being attributed to a spectrum where the step was clearly visible at the correct position, showing few features before and after the step.

**Figure 3:**
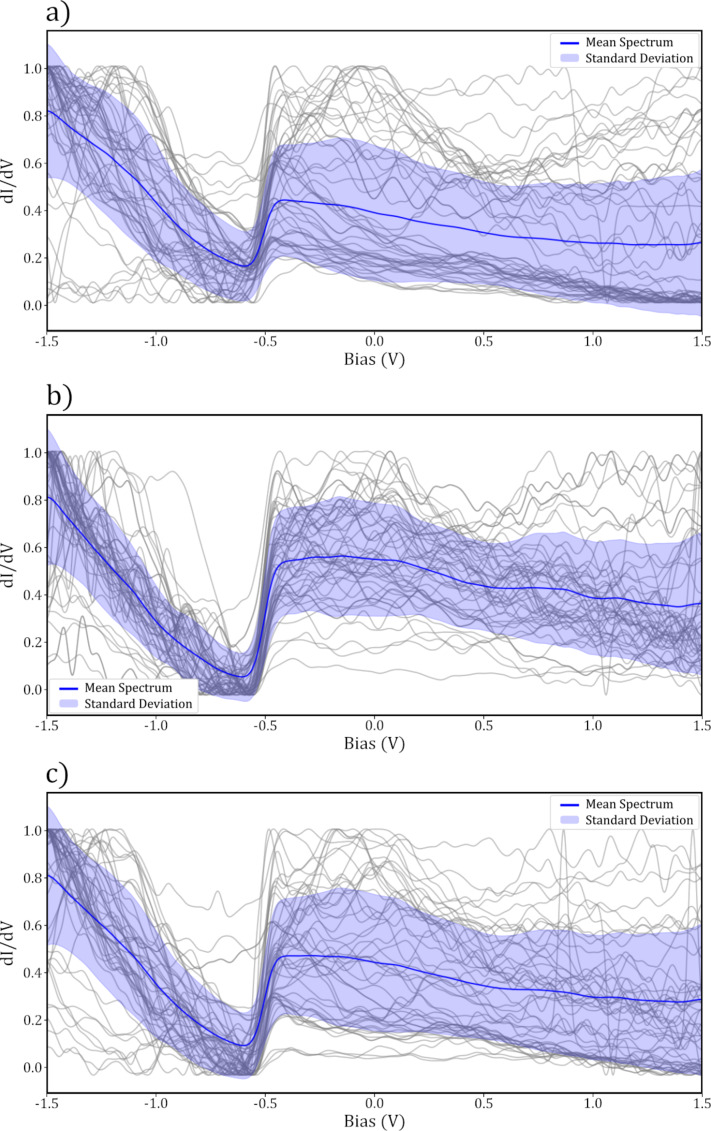
Samples of 50 normalised spectra (grey lines) taken over the clean Au(111) surface, mean (blue line) and standard deviation (shaded area) for (a) surface state “step visible”, (b) surface state “good” and (c) binary “good” labels.

**Figure 4:**
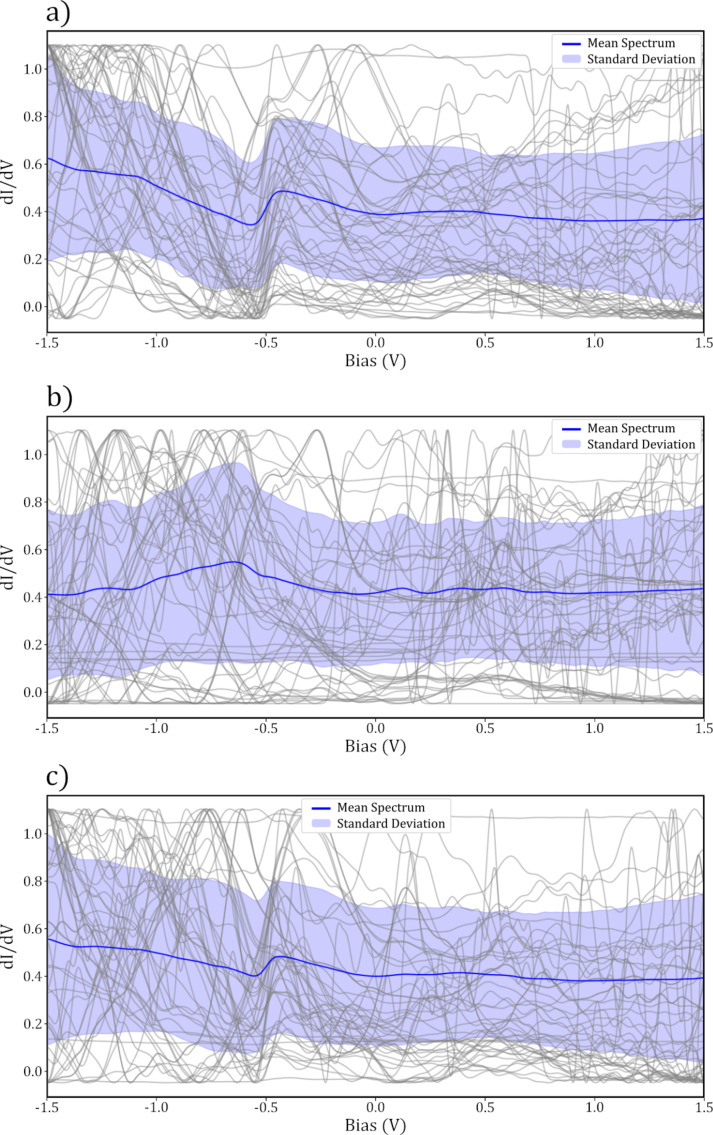
Samples of 50 normalised spectra (grey lines) taken over the clean Au(111) surface, mean (blue line) and standard deviation (shaded area) for (a) surface state “peak visible”, b) surface state “not visible” and (c) binary “bad” labels.

Whilst it was clear which data fell into each category, we note that even the data with the most visible surface state contained a background slope, as seen in Figure 3c. It is well understood that different suitable STS tips can produce considerable variation in the features observed in spectra taken over a bare substrate, including slopes through the bias range [10,20]. Therefore, the classification of what constitutes as a “good” tip is in a sense somewhat arbitrary and dependent on what the end user is interested in investigating. For the purposes of this work, we chose the primary point of interest for classification as the visibility of the surface state step at the correct bias. Our data were therefore classified with this trend in mind, with the final “good” classifications often containing a trend in the region below −0.5 V. The SS “step visible” label was given to spectra whose curves show the step in the correct position, but where a general trend (slope) was also visible throughout the data. The SS “peak visible” label applied to spectra where there was an apparent feature at the correct position for the step, but not necessarily a step, and the final SS “not visible” label was given to spectra where no feature resembling the surface state could be observed. A representative sampling of spectra from each labelling category are shown in Figure 3 and Figure 4, with all spectra obtained shown in Supporting Information File 1, Figure S2.

For the final classification, the SS “good” and SS “step visible” categories were combined into a single “good” category, and the SS “not visible” and SS “peak visible” were combined into a “bad” category. This was done to allow for a simple binary classification on the basis that further distinction between the classes is unlikely to improve the final model and would greatly increase the complexity of the problem.

Table 1 shows the number of spectra in each category after the labelling step. For ML training, the data were split into training, validation, and test sets at a ratio of 70:10:20. This left 1823 spectra for training, 260 for validation, and 521 for final testing.

**Table 1:** Number of spectra in each category.

Labels	Count

SS “good”	384
SS step visible	482
SS peak visible	1169
SS not visible	569
binary “good”	866
binary “bad”	1738

### Classification methods

#### Machine learning classifier

With the labelling completed, it was possible to train a series of 1D convolutional neural networks (CNNs). In total, 72 models were trained, varying the number of convolutional layers between 1 and 3, the number of dense training layers between 1 and 3, the number of kernels in the first convolutional layer (32 and 64 kernels were used, doubling in successive layers), kernels of sizes 3 × 3 or 5 × 5, and dropout layers with rates of either 0.3 or 0.5, including all combinations of these. The training was carried out on the training dataset containing 1823 spectra, validating the model after each epoch on the validation set of 260.

After training, each of these models were evaluated on a test set of 521 spectra, with their final accuracies, precisions, and recalls compared. We note the recall is defined as the percentage of all data labelled as “good”, which is then also classified as “good”. This metric therefore places more weight on false negatives than the precision metric and is also not as largely skewed by imbalanced datasets as the accuracy metric.

The model which achieved the best balance between the three metrics was one which contained two convolutional layers starting with 5 × 5 kernels, 32 in the first layer and 64 in the second, a single dense training layer, followed by a dropout rate of 0.3. The architecture of this model is shown schematically in Supporting Information File 1, Figure S1. This achieved an overall accuracy of 86%, a precision of 85%, and a recall of 70%.

#### Deterministic classifier

For the deterministic classifier, we required a method which is able to adapt to the entire dataset with a clear set of rules, outputting a metric describing how close any individual spectrum is to an idealised surface state spectrum. To this end, we implemented a simple model to calculate the difference between the surface state step at −0.48 V with a perfect step function, both normalised between 0 and 1.

In principle, for an “ideal” metallic tip, the step function would be clearly visible within the spectrum at the correct bias. However, as noted above, the majority of the data we acquired were not completely flat and showed a noticeable slope even when the SS was clearly visible. Therefore, in order to make a comparison between these tips and the ideal step function, additional processing is needed.

**Figure 5:**
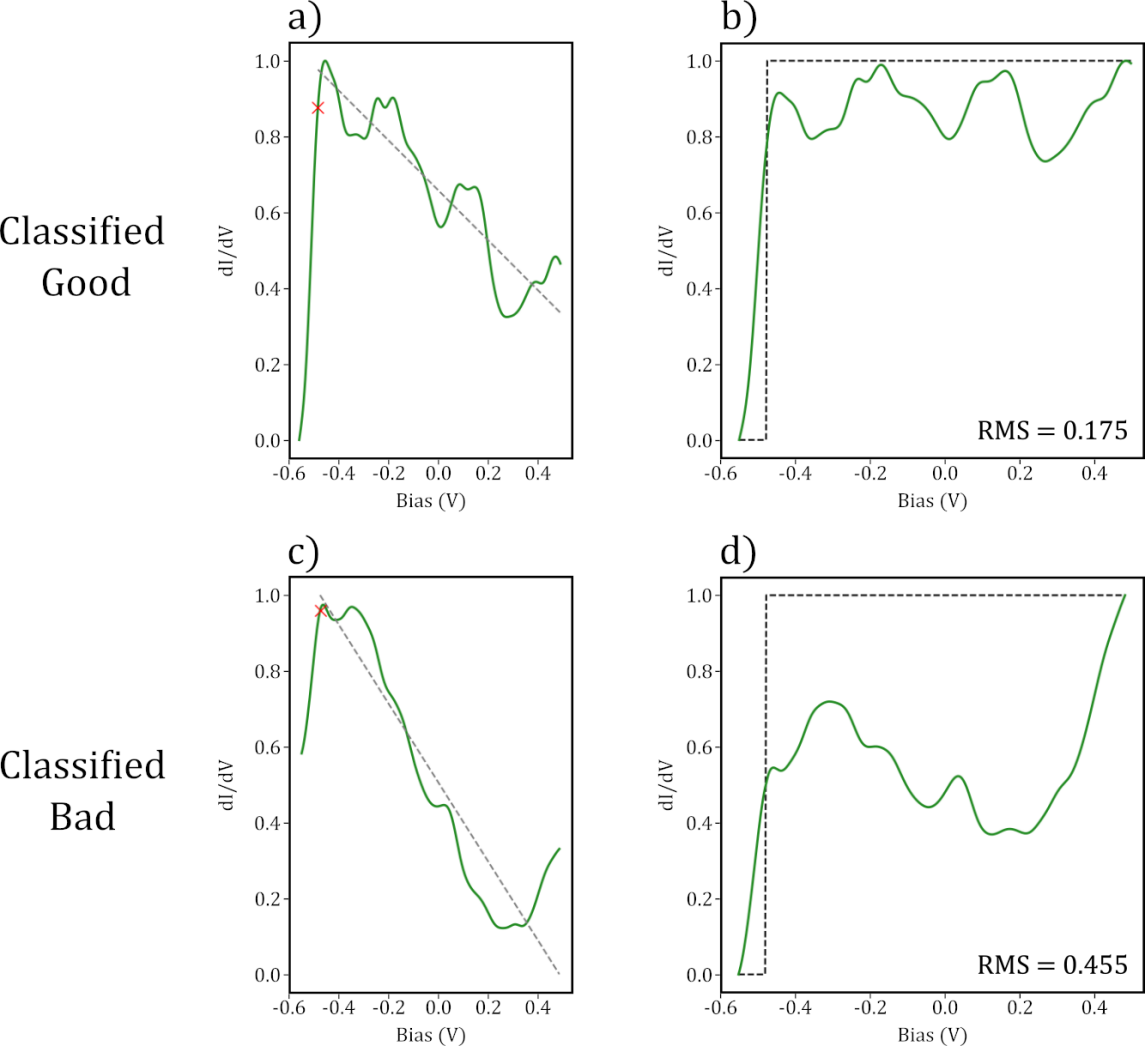
(a) and (b) show the categorisation window on a “good” and “bad” spectra, respectively. The red crosses show the automatically located turning point of the step, and the dashed gray lines show the linear fit found past the step. (b) and (d) show the spectra in (a) and (b) with their respective linear fits subtracted. The black dashed curves show the ideal surface state step function.

First, the spectra were cropped to remove features outside of the categorisation window, which for this dataset was the bias range from −0.55 to 0.5 V. From here, any general trend/slope visible in the data needs to be found and subtracted from the step. Commonly in our data, it seems that the trend is a linear offset in the 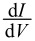, and hence a linear function after the step can be fit to the data, and then subtracted from the original spectrum. The specific location of the turning point of each spectrum is obtained by finding the minimum of the differential of the curve within a small range around −0.48 V, and the step is assumed to be contained within the categorisation range, following this determined turning point. From this, a linear function is fit to the window, an example of which is shown for both a “good” and “bad” classified tip in Figure 5a and Figure 5c, respectively.

Once the linear trend is found, it is subtracted from the original spectrum, the result of which is shown in Figure 5b and Figure 5d. For a “good” spectrum, the resultant curve should appear roughly as a step function, and so by direct comparison to a perfect step function, starting at the turning point found earlier, a deterministic classification measure can be made. The specific metric output as the difference between these two curves is the root mean squared (RMS) error between the two, which is described by Equation 2:


[2] $$\text{RMS}=\sqrt{\sum_{i=1}^{n}{\left({\hat{y}}_{i}-y_{i}\right)}^{2}}.$$

**Figure 6:**
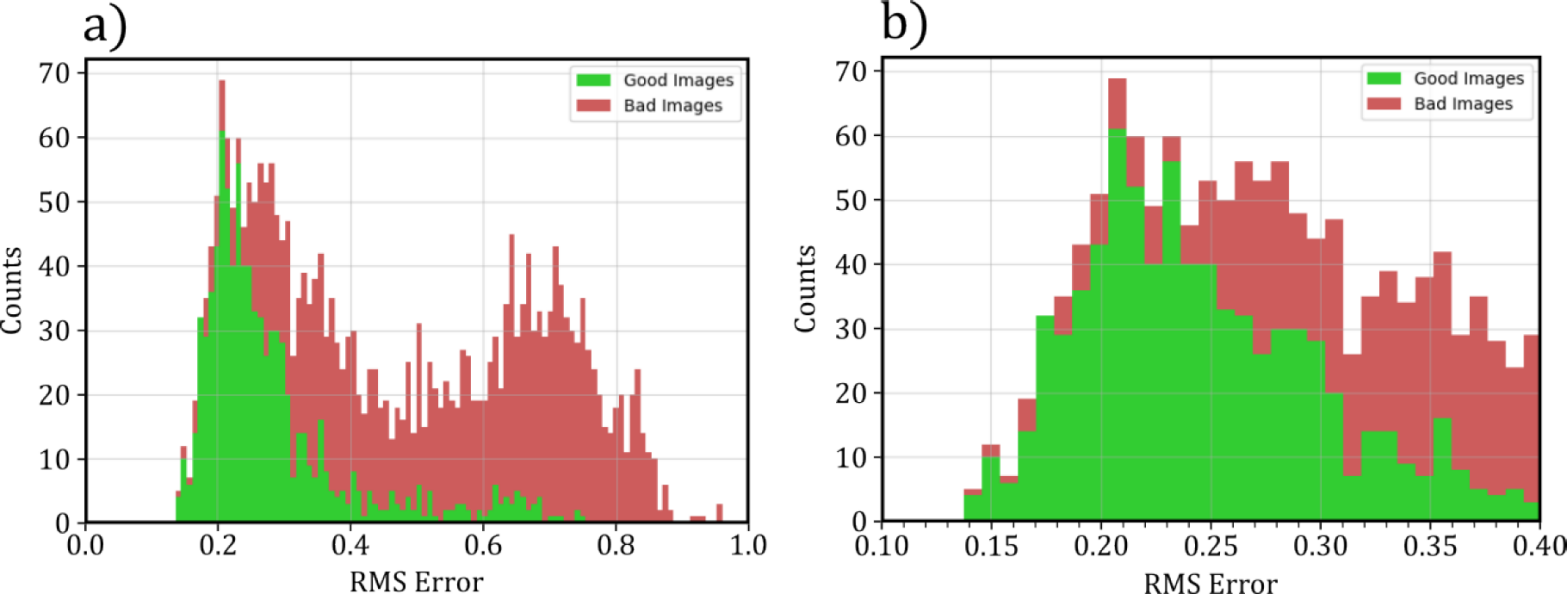
Stacked histogram made from labelled spectra, calculating the RMS error between each processed spectrum and an ideal step function. (a) shows the full range of RMS, with (b) showing the values between 0.1 and 0.4.

Here, 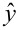 are the perfect step function data points, and *y* are the spectra for classification. To evaluate the optimal thresholds for classification, a stacked histogram was plotted, showing the spread of the RMS in each category. This histogram is shown in Figure 6. From this, the final threshold was chosen *<*0.25, with all spectra resulting in a value within this range classified as “good”.

Using this method, the deterministic model was able to achieve an overall accuracy of 82%, a precision of 86%, and a recall of 53% when evaluated on the same test set used for the ML model.

## Results and Discussion

Both the deterministic and ML-based models were tested on the same isolated test set of 521 spectra, with the final results as given in Table 2. Both models achieve very similar accuracies and precisions; however, the recall for the deterministic model is significantly lower than in the ML model. In practise, this lower recall would mean that more tips which a human may classify as “good” would be misclassified as “bad”, slowing down the overall tip preparation process. However, since the precisions of both the ML and deterministic models are very similar, the probability of an automated tip preparation script exiting with a “bad” tip would be roughly the same using either model. Since both models show comparable results in the precision of the final classification, the main advantage to using the deterministic model over ML is that the classifier requires much less labelled data for its creation, and hence is easier to apply to a new system.

**Table 2:** Table showing the accuracy, precision and recall obtained using deterministic and ML models to classify probe tips based on spectroscopy measurements.

	Deterministic	ML

accuracy	82%	86%
precision	86%	85%
recall	53%	70%

Our ML accuracies are consistent with the prior work undertaken by Wang and colleagues [15]. Their highest ML-based classifier was able to achieve a precision of 84% and a recall of 74%, whereas our DM-based results are substantially better than the DM approach they trialled, which used correlation-based metrics and only achieved a final precision of 41% and a recall of 53% (no accuracies were given for this work).

It should be noted, however, that Wang et al. attempted to make a classification between five different labels of spectra, whereas our dataset was split into a binary “good” or “bad” before training. In general, binary classifiers are expected to achieve higher accuracies as the differences between the two categories are less subtle.

### Automated experiment discussion

In addition to automatically classifying the tip quality via STS on the Au(111) substrate, the script automatically located each SnPc molecule on the Au(111) surface, identified the different configurations of the molecule, and carried out lock-in 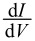 measurements over the centre of each. In this section, we will discuss the STS data taken on the molecules, discuss the impact on the STS data quality due to the quality of the tip and highlight the advantages of automated assessment of tip quality and statistical categorisation of the data in STS.

### Molecule location and identification

Once a series of Au(111) surface spectra had been obtained for use in the classifier training, the script continued to obtain STS measurements over the centre of each located SnPc molecule, whilst also distinguishing between the two configurations (SnUp and SnDown) prior to measurement. The identification of each molecular configuration was determined using the CC method with two separate reference images as shown in Figure 1d,e.

For the final distinctions on the Au(111) surface, the CCR thresholds used for the SnUp and SnDown molecules were 0.983 and 0.980, respectively. Using these thresholds on a small test set of 13 images, the script was able to locate the positions of SnUp molecules with 100% accuracy and precision, whereas on the SnDown molecules the accuracy achieved was 95% with a precision of 96%.

Once located, lock-in 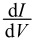 curves were obtained over the central atom of each molecule located, using the same range of −1.5 V to 1.5 V as for the bare surface.

### Note on SnPc switching instability

The adsorption of SnPc on coinage metal substrates is well studied, and the molecule is known to undergo an irreversible switch from the SnUp to the SnDown state on the Ag(111) surface [21] under hole injection. This is usually carried out intentionally by positioning the tip over the centre of an SnUp molecule, and applying a bias pulse via the tip of less than −1.9 V. On injection, the Sn atom within the molecule is transiently oxidised to Sn^3+^, which favours a new position closer to the surface, where the atom binds to the Ag(111), at which point charge transfer from the substrate to the molecule will return it to its neutral state [21].

Whilst carrying out bias spectroscopy over these SnPc molecules, it was found that a switch could occasionally be induced, even if the bias range used did not reach −1.9 V. With moderate negative bias (e.g., −1.5 V) a switch would commonly occur, and even with parameter adjustments to reduce the probability of switching (i.e. reduced integration times), there was still a chance that the switch would be induced, as can be seen in Supporting Information File 1, Figure S4.

In the automated experiment, an image would be taken, the molecules located based on this image, and then spectra would be obtained, saving each spectrum with a label indicating which configuration of molecule the spectrum was taken over. Unfortunately, due to this switching occurring over specifically the SnUp molecules, spectra labelled SnUp had the potential to be unreliably labelled. Additionally, it was observed that these switches could occur at the start of the measurement in the initial setting of the bias, or during the bias sweep itself, meaning identifying if a switch had occurred could not be reliably inferred from simple analysis of the STS spectrum. For this reason, the results shown in the next section will only consider spectra taken over the SnDown configuration, as the labelling of these was reliable.

### Results

Using our STS spectra surface state classification method as described previously, we were able to process the entire dataset collected and to categorise the spectra taken on molecules as being acquired with either a “good” tip, or with a “bad” tip.

**Figure 7:**
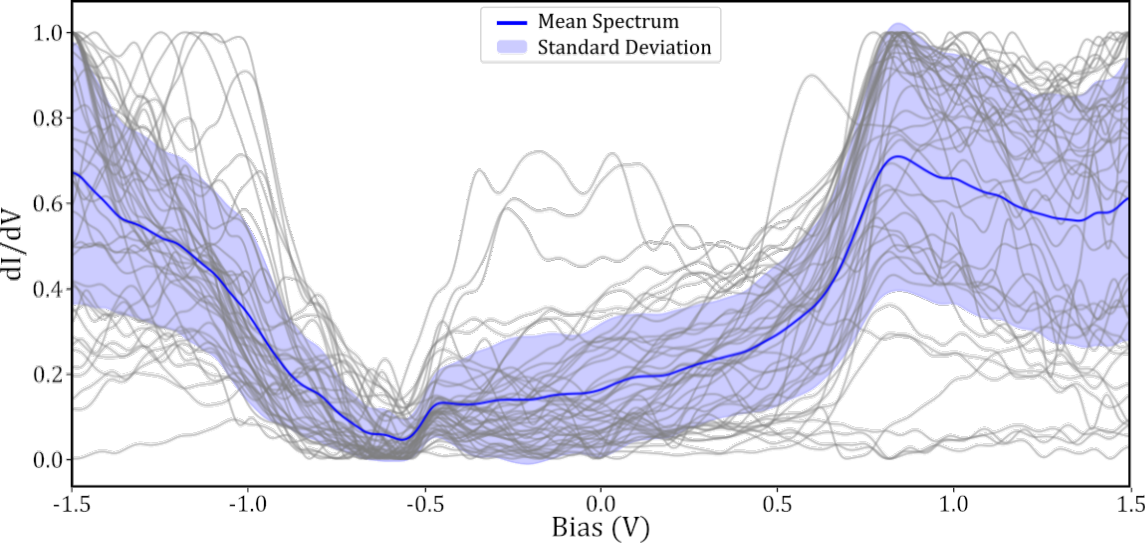
Gray curves show 49 normalised STS measurements taken over the centre of SnDown molecules taken with a “good” tip. The blue curve shows the mean and the standard deviation is shown in shaded purple.

Throughout the data gathering, a total of 86 images (and so probe tips) passed the *I*(*z*) and CCR pre-classification steps and were used to obtain molecular STS measurements. Of these 86 tips, 30 were classified as “good” and 56 as “bad”, based on the analysis of the final STS spectra on Au(111). These 30 “good” tips were used to obtain spectra over a total of 49 SnDown molecules. The mean of these curves is shown in Figure 7, where it can be clearly seen that there is a peak at roughly 0.8 V, which is not present in the bare surface spectra seen in Figure 3c.

Previous STS data of SnDown molecules show a clear increase in conductance at both −0.85 and 0.75 V when imaging at a setpoint of 50 pA [22]. These peaks in conductance correspond to the lowest unoccupied molecular orbital (LUMO) and highest occupied molecular orbital (HOMO), respectively. The cited work, however, was carried out on the Ag(111) surface, as opposed to Au(111) used here, which could explain the slight shift in the position of the HOMO from the literature value of 0.75 V to our consistent measurement of roughly 0.8 V. In addition, the work also suggests a current dependence on the position of the HOMO, which could be a contributing factor to the difference.

When comparing the “good” 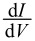 spectra taken over the molecules (Figure 7) to the binary “good” bare surface spectra (Figure 3c), the surface state and general increase in conductance at biases below −0.5 V can be seen in both. However, unlike the HOMO, which is clearly visible in the molecular 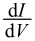, the LUMO is not visible at the expected bias value of −0.85 V. This is possibly due to the peak being obscured by the shoulder in the negative portion of the spectra. Comparing the region between −1.5 and −0.5 V in both spectra, it can be seen that the mean curve for the molecular spectra contains an additional feature which is not present in the mean bare surface state spectrum. The features contained within this could contain the LUMO, but this is difficult to ascertain without completely deconvolving the tip and sample LDOS.

**Figure 8:**
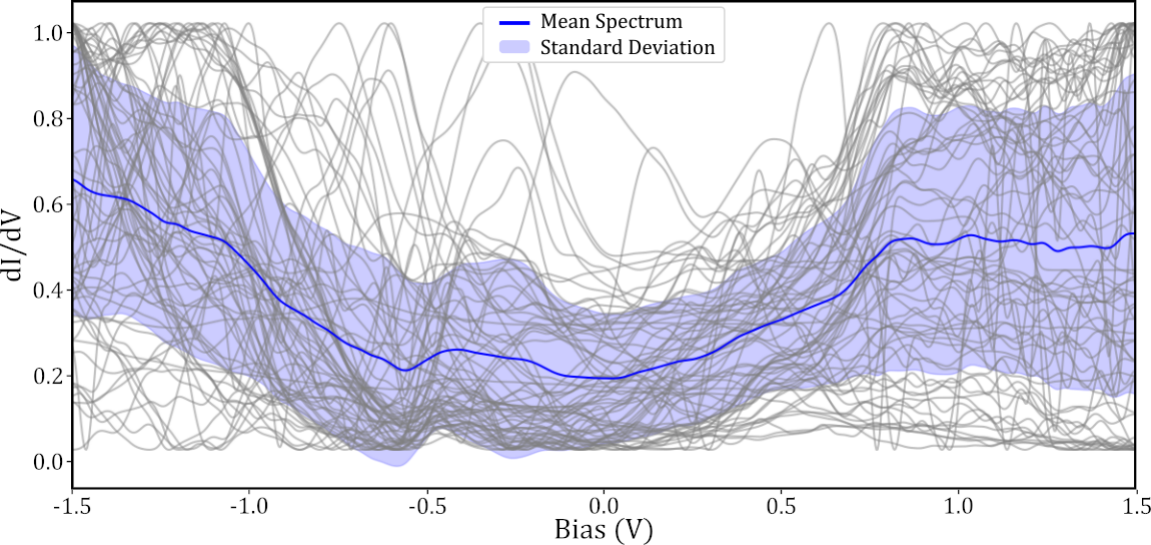
Gray curves show 71 normalised STS measurements taken over the centre of SnDown molecules taken with a “bad” tip. The blue curve shows the mean and the standard deviation is shown in shaded purple.

Figure 8 shows the mean and standard deviation of the 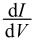 spectra taken over SnDown molecules taken with a “bad” tip. By comparing this to Figure 7, it is clear that the HOMO peak at 0.8 V is much less prominent. In addition to this, the features throughout the spectra have become less evident. This clear difference between the molecular STS taken with a “good” and a “bad” tip, with the former showing expected features, reinforces that the tip state classification was successful in producing higher quality spectra, and highlights the importance of appropriately charactering the tip state before STS experiments.

A clear advantage to performing automated experiments with a large number of different tips and over a large number of molecules in different surface positions is that, statistically, variations in the spectra due to the changes in the tip or small changes in the molecular adsorption, will be averaged out, and better approach those from ensemble techniques. As can be seen in both Figure 7 and Figure 8, there is a substantial variation in the individual spectra around the mean curve. This is most likely due to variations in the quality of the tip or slight differences in the molecule itself. However, with a large enough aggregate of different tips, and taking STS measurements over different molecules, when averaged, these small variations should be dominated by the consistent features present in all the data. This can be seen particularly well in Figure 7, where some of the individual molecular spectra (grey curves), which here were all taken with tips classified as “good”, show a featureless region around the HOMO, whilst others clearly show a strong peak. With individual spectra, it is possible that specific features in the 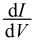 could remain unobserved due to spurious problems with the tip.

For a human operator, taking a large number of spectra, with different, yet still “good” tips, on different instances of the same molecule is extremely time-consuming. However, with the entire process being automated, this can be carried out very simply, and without any need for constant monitoring.

**Figure 9:**
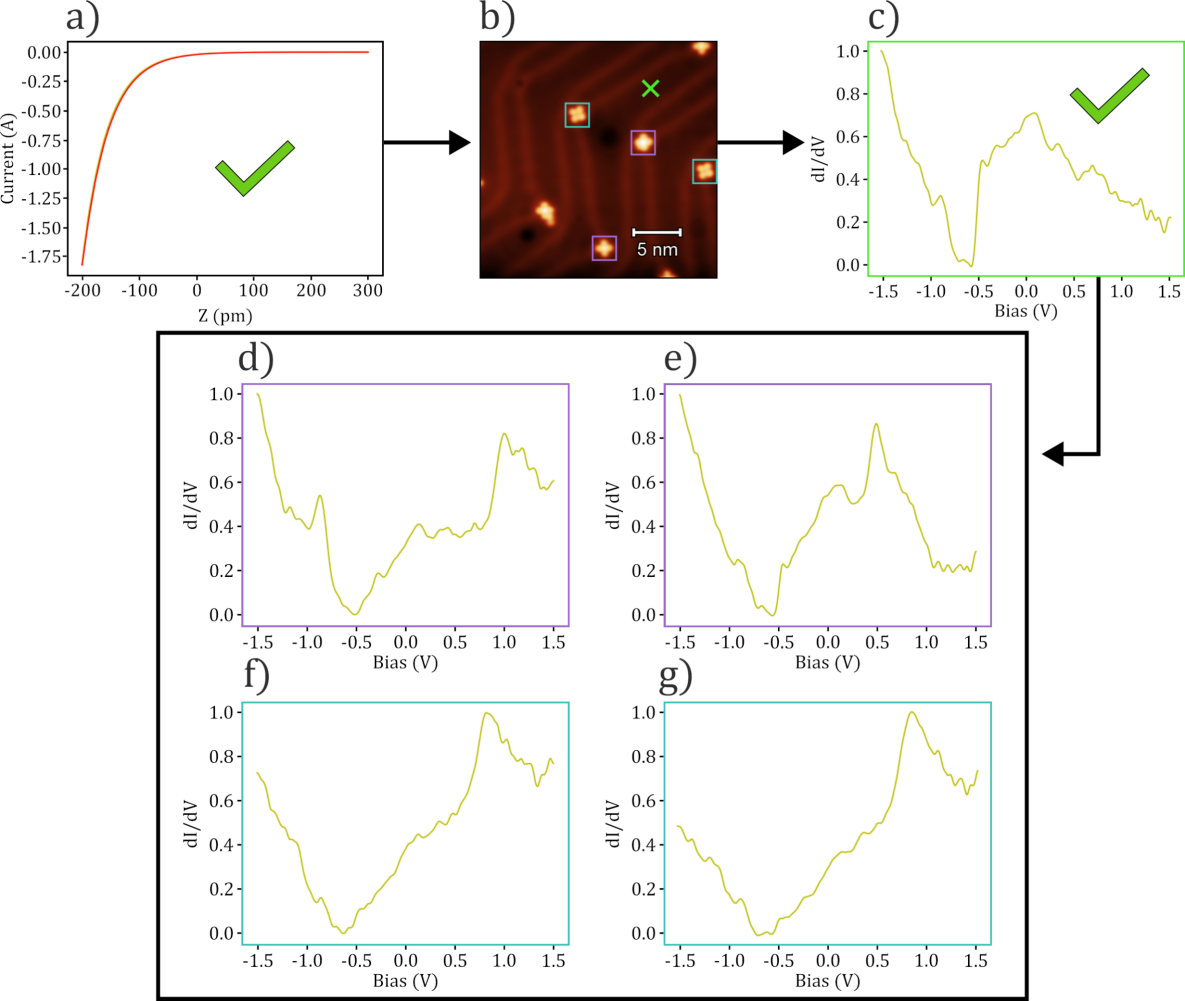
Example flow of an automated spectroscopy experiment taken over various SnPc molecules on the Au(111) surface. (a) An initial *I*(*z*) measurement is taken, where an exponential dependence is observed and so moves onto imaging, (b). The tip is then classified to be “good” based on imaging, and so a clean area of the substrate is located (marked by a green cross), where a surface STS measurement is taken, (c). This is then classified to be “good”, at which point the various orientations of SnPc are located (SnUp in pink boxes and SnDown in blue boxes), where STS measurements are taken as shown in (d–g). (d) and (e) correspond to measurements taken over SnUp molecules, while (f) and (g) correspond to SnDown. The script would then change the tip and repeat the steps, over different areas, varying the tip after each set of STS measurements (formed through in situ tip preparation).

We note that while we collected data using both types of tip in order to highlight the differences in quality, in a real use case data collection would be improved by using the proposed method of classifying the tip based on a 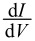 spectrum taken over the bare surface, such that the script would only take molecular spectra using probes which have been classified as “good” based on the surface state spectra. An example flow diagram with data taken from a generation run where the surface state spectrum was classified as “good” is shown in Figure 9.

## Conclusion

We have shown that it is possible to perform a fully automated experiment, carrying out STS measurements over targeted areas of specific organic molecules, including the ability to modify and characterise the state of the tip, by both analysis of its spectroscopic characteristics and imaging quality, without the use of machine learning. This enables the ability to obtain a large number of spectra over various features on a surface, with a variety of characterised tip states, without the need for an operator to be present, and to perform statistical analysis of the spectroscopic data, via the automated labelling of the state and the location of the spectrum.

Importantly, the ability to carry this out without machine learning means that this method can be easily adapted to different adsorbate/substrate systems without the need for extensive data collection to train ML models. This methodology can aid in the rapid characterisation of new materials via automated probing of different features in a system, taking numerous measurements over different areas, only requiring an operator once the experiment is complete, to process the resultant data for analysis.

## Supporting Information

File 1 Additional Information.

## Data Availability

Data generated and analyzed during this study is available from the corresponding author upon reasonable request.
