# Supplementary material for: Automated collection and categorisation of STM images and STS spectra with and without machine learning
Source: Beilstein J Nanotechnol. 2025 Aug 18;16:1367–79. doi: 10.3762/bjnano.16.99 (PMC12415897; doi:10.3762/bjnano.16.99)
Supplement: File 1 — Additional Information. [file Beilstein_J_Nanotechnol-16-1367-s001.pdf]

BEILSTEIN JOURNAL OF NANOTECHNOLOGY

Supporting Information

for

Automated collection and categorisation of STM images and STS spectra with and without machine learning

Dylan Stewart Barker and Adam Sweetman

Beilstein J. Nanotechnol. **2025,** *16,* 1367–1379. doi:10.3762/bjnano.16.99

Additional Information

License and Terms: This is a supporting information file under the terms of the Creative Commons Attribution License (https://creativecommons.org/licenses/by/4.0). Please note that the reuse, redistribution and reproduction in particular requires that the author(s) and source are credited and that individual graphics may be subject to special legal provisions.
The license is subject to the *Beilstein Journal of Nanotechnology* terms and conditions: (https://www.beilstein-journals.org/bjnano/terms)

Supporting Information

for

Automated collection and categorisation of STM images and STS spectra with and without Machine learning

Dylan Stewart Barker*[1] and Adam Sweetman*[1]

Address: [1]School of Physics and Astronomy, University of Leeds, Leeds LS2 9JT, United Kingdom

Email: Dylan Stewart Barker - D.S.Barker@leeds.ac.uk; Adam Sweetman - A.M.Sweetman@leeds.ac.uk

* Corresponding author

Supporting Information

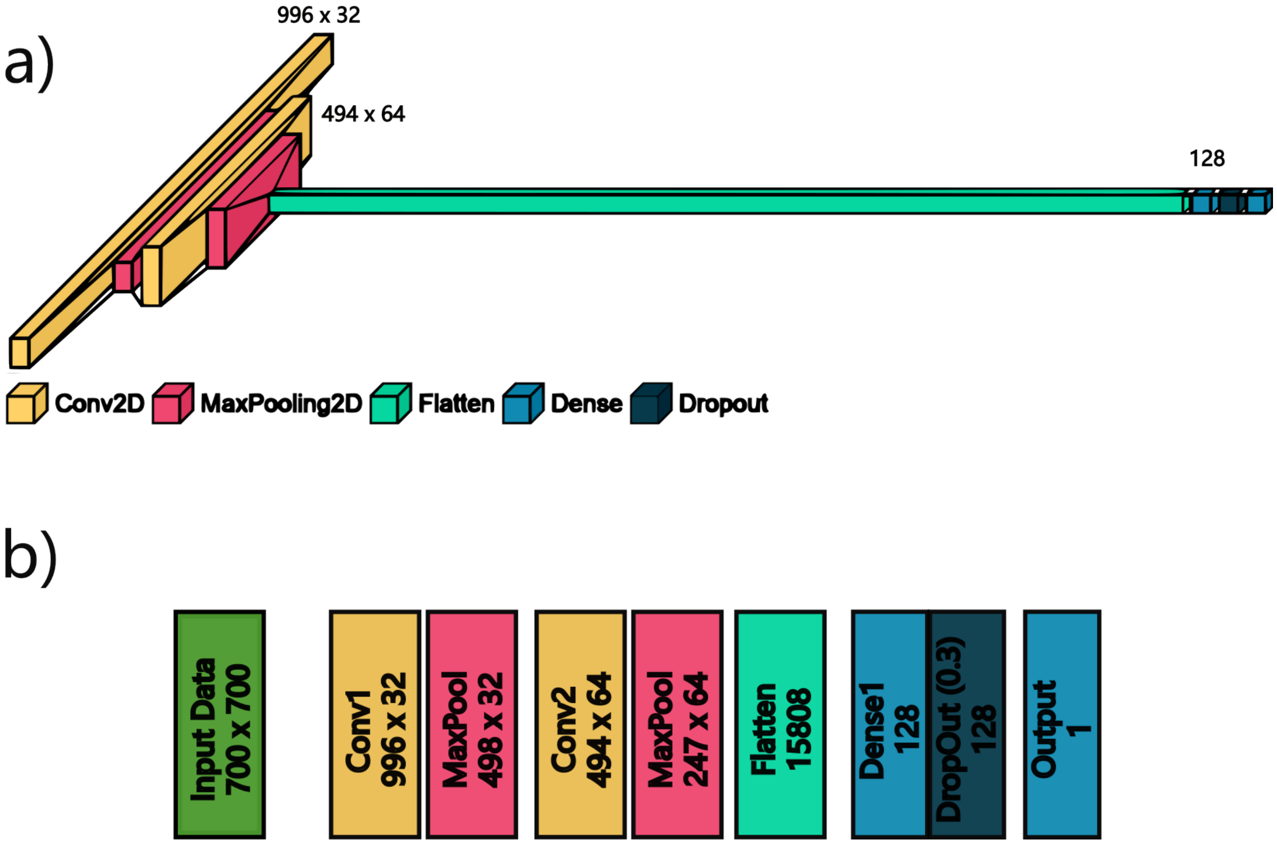

Figure S1: Architecture of the CNN model used to classify STS spectra. It consists of a total of 8 layers: 2 consecutive convolutional-pooling layers, a flattening layer, a single training-dropout layer, and ending with a single dense output layer.

Upgraded Rotation Optimiser

SnPc molecules can adsorb in multiple orientations on a surface, resulting in the basic feature finding used in Barker *et al.* [1] not locating these molecules accurately. Because of this, additional steps are needed in automatically finding the exact orientation of the molecules on the surface. If the reference image is used to calculate the CCR without an applied rotation, it will not fully represent the similarity of the tip state with that used to obtain the reference image. It was found that the polar translation method described previously [1] was not able to accurately find the rotation in all cases, therefore, the rotation optimiser needed to be improved.

The improved method used here calculates the CCR of the input image based on a series of rotated

reference images, starting with a coarse approach of 6 references each rotated from each other by 60°. The highest correlation of these references is then rotated again by ±30° in steps of 10°, followed by a finer step of rotating the maximal rotation by ±5° in steps of 1°. This results in a rotation alignment with an angular resolution of 1°.

This method was carried out in addition to the original polar translation method, taking the angle which resulted in the highest CCR between the two used as the optimal rotation.

I(z) Classification

In addition to the CC based image classification, I(z) spectroscopy measurements are taken and classified before and after the topography scan, in order to filter out the worst tips. Checking if an I(z) for a specific tip shows an exponential trend is a very efficient filtering step, removing bad tips without the need to obtain an entire scan, reducing the time needed for these classifications from ≈ 4 minutes to ≈ 10 seconds. We note that while a tunnelling junction does not mean a tip will image well, or is necessarily metallic, a non-tunnelling junction reliably indicates a contaminated tip.

The classification of this exponential dependence was performed using the pearsonr function from Scipy in Python. This function evaluates the linear relationship of an input set of data, so by taking the logarithm of the current channel of the I(z) and inputting this into the pearsonr function, a metric for how close to a perfect linear relationship can be calculated. The pearsonr function uses the following equation:

$$r = \frac{\sum(x - m_x)(y - m_y)}{\sqrt{\sum(x - m_x)^2 \sum(y - m_y)^2}} \tag{S1}$$

Where r is the output measure of the linearity of the input data, x is the distance or z vector, y is the logarithm of the current vector, $log(I)$, and m_x and m_y are the mean values of x and y respectively. The output of this function is a number between 0 and 1, where 1 corresponds to a perfect linear function. Using a threshold of > 0.9995 for a good linear (and therefore exponential) trend, spectra

were classified with an overall accuracy of 94% and a 100% precision after being tested on a small set of data containing 36 total spectra.

Unstable or generally bad tips will show a non-exponential trend in their I(z) curves, with either jumps as the tip changes in structure throughout the measurement, or simply a different trend which usually indicated that the current is flowing through a non-vacuum junction, i.e., the tip is in constant mechanical contact with the surface. Since the CC classification needs a full scan for classification, it is much quicker for a single I(z) curve to be taken and analysed rather than a topograph. With this in mind, applying an I(z) classification as a filtering step should increase the efficiency in all automated STM tip classification methods. Examples of good and bad tips are shown in Figure

I(V) Generation

The classification of $\frac{dI}{dV}$ spectra on the Au(111) surface requires that spectroscopy measurements are taken over an area free of contaminants or other defects. Under these conditions, measurements should show a characteristic step at a bias of around -0.48 V. Before being able to test the automation of this classification by ML, a large amount of data is needed for model testing. Therefore, this script first analyses a given topograph to locate the largest area of bare substrate, over which the I(V) measurements can be taken. Once found, 15 individual I(V) measurements were taken in this area, spread out in a grid to ensure measurements were taken over varied positions on the surface.

The I(V) curves were taken in the range -1.5 V to 1.5 V using the lock-in method, with 1000 points of measurement taken in this range. These parameters were chosen as it is conservative enough to keep the risk of a tip change due to the measurement low, whilst still broad enough to capture a typical bias window for STS of organic molecules on this surface.

a)

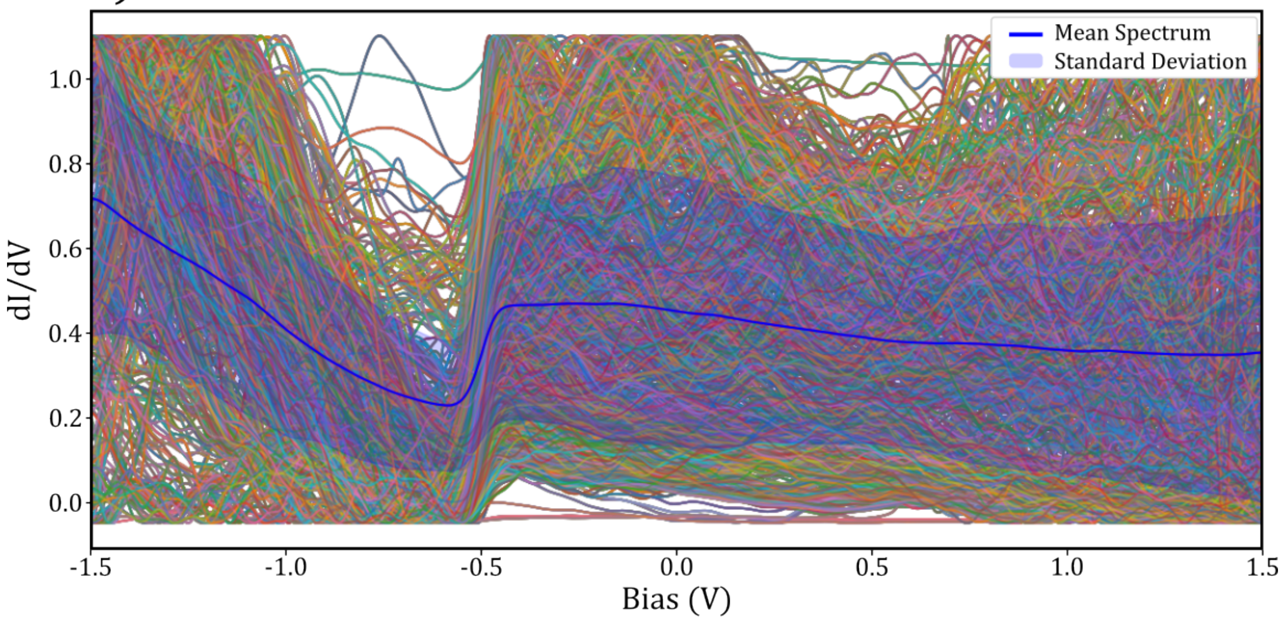

b)

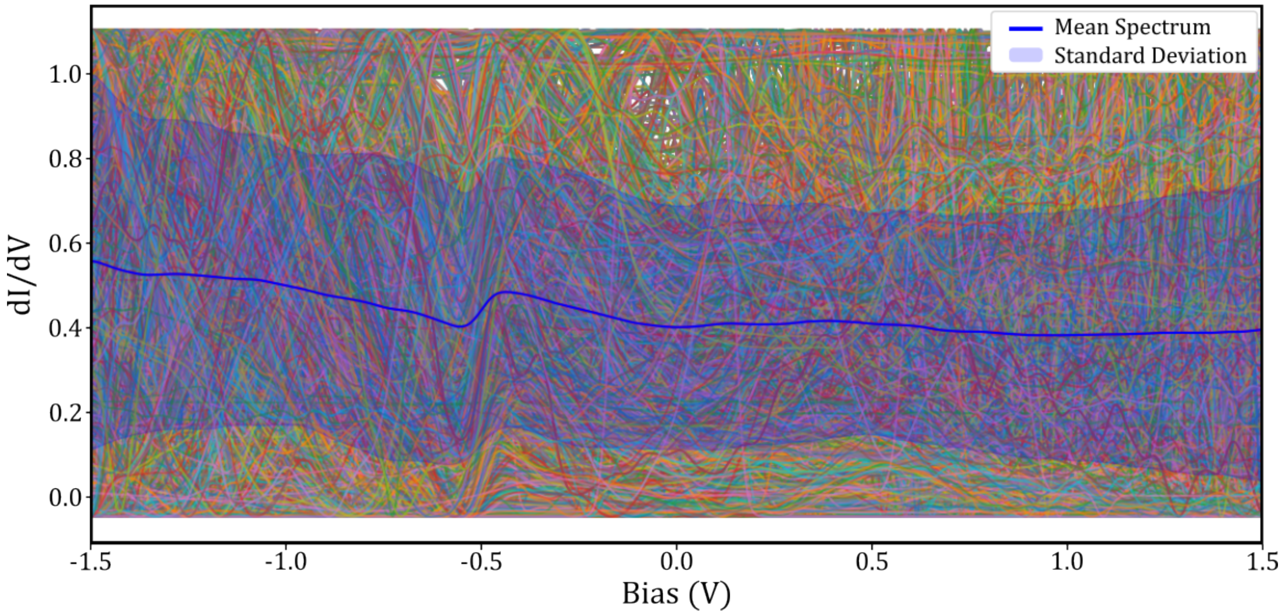

Figure S2: All normalised spectra, mean (blue line) and standard deviation (shaded area) for a) Binary "good" and b) binary "bad".

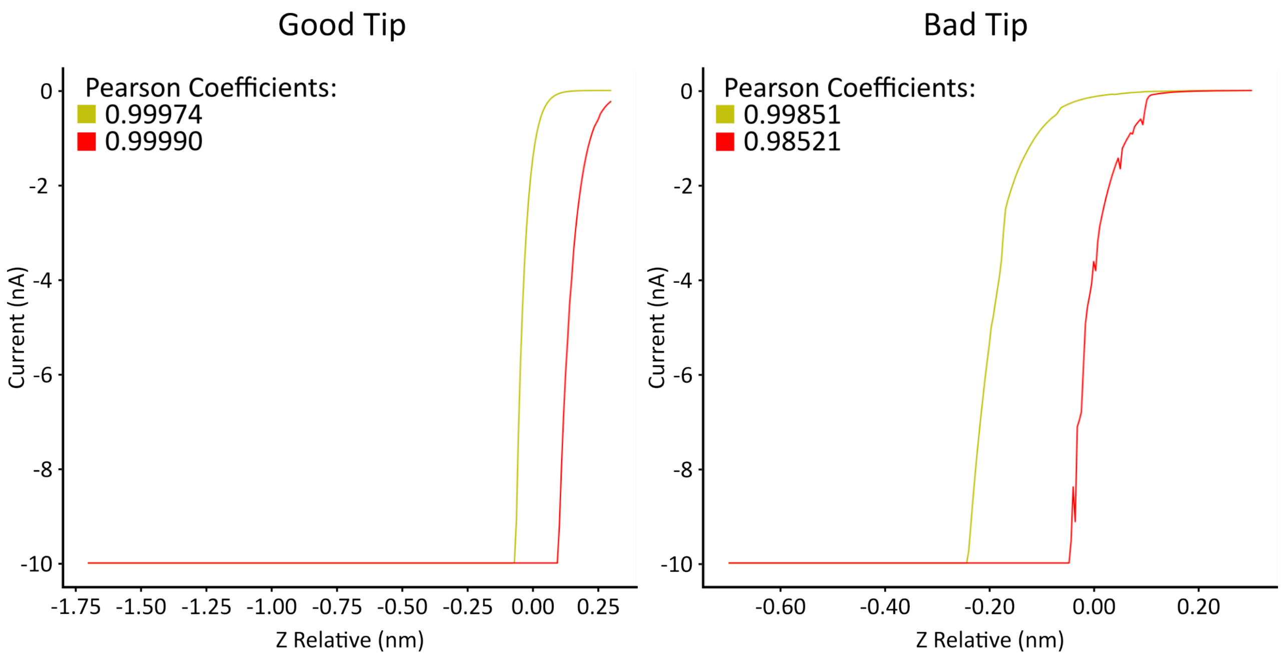

Figure S3: Example I(z) spectra for a good tip (left) and a bad tip (right) with the corresponding pearson coefficients.

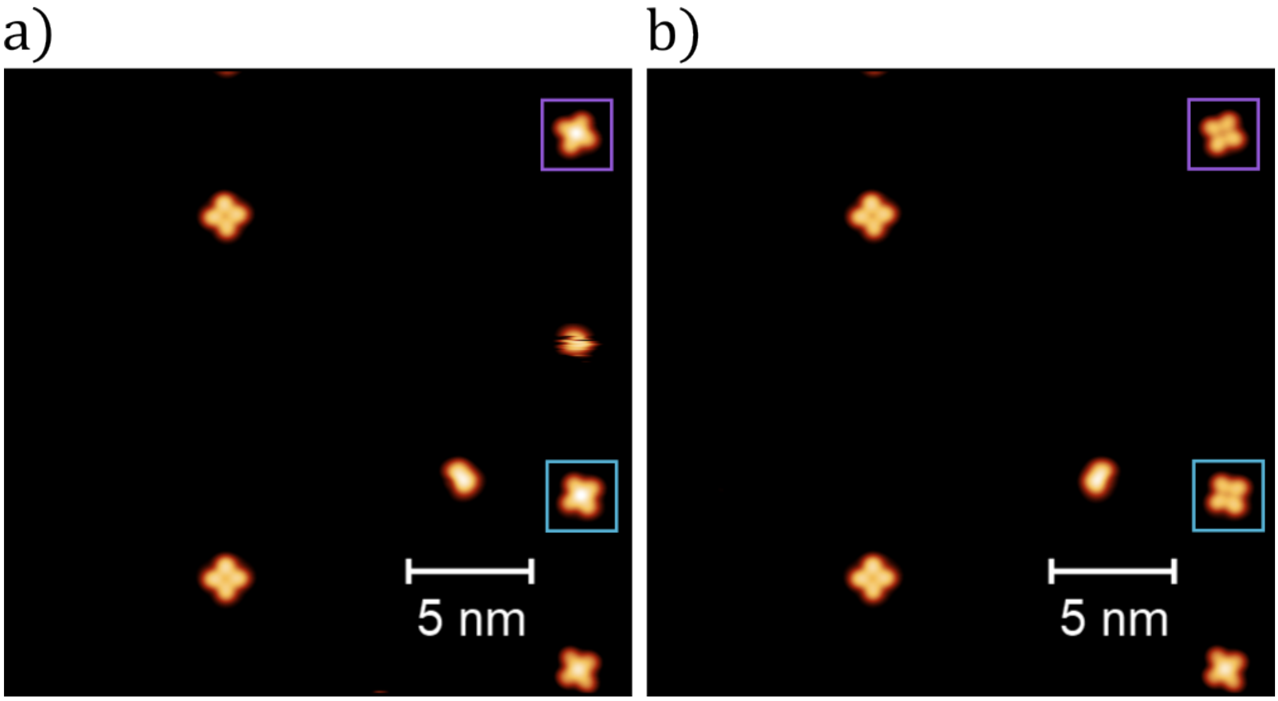

Figure S4: Low coverage of SnPc on Au(111) taken at 5 K, 100 mV, 20 pA. a) shows an initial image taken, after which STS measurements between −1.5 and +1.5 V are taken over the molecules. b) shows the same area after some of these measurements have been taken, blue and purple squares show the same molecules which have switched from SnUp to SnDown. Note the SnUp at the lower edge of the image has not switched, despite undergoing the same spectroscopy range as the other two, showing the spurious nature of this switching.

References

1. Barker, D. S.; Blowey, P. J.; Brown, T.; Sweetman, A. *ACS Nano* **2024**, *18*, 2384–2394. doi:10.1021/ACSNANO.3C10597.
